# New-Onset Illness Anxiety Disorder After Helicobacter Pylori Infection: A Case Report

**DOI:** 10.7759/cureus.52613

**Published:** 2024-01-20

**Authors:** Suhail A Labban, Leen Murshid, Amal Yousef Alhazmi, Ammar Y Bafarat, Nuha M Alharbi

**Affiliations:** 1 College of Medicine, King Saud Bin Abdulaziz University for Health Sciences, Jeddah, SAU; 2 Psychiatry, King Abdulaziz Medical City, Jeddah, SAU; 3 Psychiatry, King Abdulaziz Hospital, Jeddah, SAU; 4 Psychiatry, Ministry of National Guard Health Affairs, Riyadh, SAU

**Keywords:** psychotherapy, health anxiety, hypochondriasis, h. pylori, illness anxiety disorder

## Abstract

In this study, we present a case of new-onset illness anxiety disorder (IAD) in a 21-year-old female patient after *Heliobacter pylori *infection. The patient experienced a distressing preoccupation with having or acquiring a serious illness with mild somatic symptoms for more than six months. IAD adversely affected our patient’s life and made her engage in excessive care-seeking behaviors and maladaptive avoidance in some instances. In this case, we highlight the unique presentation of symptoms related to illness anxiety disorder and *H. pylori *infection. Furthermore, we discuss the possible psychosocial factors that are considered risk factors for developing IAD. We also discuss the pharmacological and psychological treatment options for patients with such a presentation.

## Introduction

Illness anxiety disorder (IAD) is a mental disorder of elevated health anxiety involving an intense fear about the possibility of having or acquiring a serious illness [1]. The prevalence of IAD in population-based studies has been reported to be 0.04-4.5% [1]. Screening in psychiatric populations found that 1.6-3.5% of patients meet criteria for IAD. Moreover, 0.3-8.5% of patients met the criteria for IAD when screened from general medical practices [1]. Locally, a cross-sectional study in Saudi Arabia reported the prevalence of IAD among medical students to be 17% [2].

Diagnosis of IAD requires preoccupation with having or acquiring a serious illness, absence of somatic symptoms (or, if present, symptoms that are only mild in severity), an elevated level of anxiety about health, proneness to alarm regarding health status, and either excessive care-seeking behaviors or maladaptive avoidance of medical settings for six months [1]. Furthermore, a diagnostic interview for IAD should include symptoms of major depressive disorder, generalized anxiety disorder, mania, psychosis, and obsessive-compulsive disorder [1]. It is also important to inquire about the level of worry about health, any previous care-seeking behaviors and the time spent on that, which diseases are feared, and the evidence upon which the patient bases this fear. Moreover, the interview must include asking about the presence and severity of somatic symptoms, and the functional impairment and limitations [1]. The psychopathology of high-level health anxiety encompasses three main domains. The first is disease conviction, which is a belief of having a serious illness that can not be shaken by negative diagnostic testing, lack of physical examination findings consistent with a disease, or explanations of the unlikelihood of having a serious disease. The second domain is disease fear, which is the high distress when presented with any suggestion of the possibility of having an illness. The final domain is a bodily preoccupation, which is the overestimation of physiologic functions, benign bodily sensations and sources of discomfort, and physical limitations [1]. Patients’ insight regarding the pathologic nature of their worry varies. Some patients can be aware that their worry is excessive yet feel helpless to control it, whereas others can not be dissuaded from their fear of having a serious illness [1].

*Helicobacter pylori* is a very common upper gastrointestinal pathogen that colonizes mostly the antral portion of the gastric mucosa. Humans are the main reservoir and the transmission occurs via fecal/oral or oral/oral routes [3]. *H. pylori* was found to be associated with peptic ulcer disease and gastritis and may progress to gastric cancer [3]. Moreover, it affects people of all ages worldwide; however, younger populations and developing countries have higher rates of it than developed nations. Globally, it infects approximately 50% of the population [3]. In China, *H. pylori* prevalence was 44.2% [4]. In Saudi Arabia, *H. pylori* is regarded as hyper-endemic. According to a local study, 46.5% of people in the city of Jazan had *H. pylori *[5]. The cities of Jeddah and Riyadh had an overall 49.8% prevalence of *H. pylori *infection among children [6]. Furthermore, infection with *H. pylori *is strongly linked to mental illnesses, notably anxiety and depression. The prevalence of depression among *H. pylori *patients in Ethiopia was 11.9%, in Turkey 24.1%, and in Bahrain 32.1% [7-9]. A Japanese study found that there is a high risk of psychological distress or depressive mood among *H. pylori*-associated atrophic gastritis (AG)-infected females aged <50 years [10].

The gut-brain axis (GBA) is a bidirectional communication pathway between the central and enteric nervous systems that connects the brain's emotional and cognitive centers with peripheral intestine processes. *H. pylori *may alter the mood regulations through interactions in this GBA [11,12]. Moreover, it has been hypothesized that the vagus nerve allows for communication between the microbiota in the gut and the brain [11,12]. Another route for this bacteria to alter mood is through the hypothalamus-pituitary-adrenal (HPA) axis. *H. pylori *produces chronic stress, resulting in a maladaptive rise in cortisol levels, which leads to mood disruption [13].

## Case presentation

A 21-year-old single female patient, not known to have any medical conditions, presented to the outpatient clinic after a referral by a family medicine physician for an assessment for panic disorder and anxiety symptoms. The patient came with her father, and she seemed reliable. She preferred that her father stay with her during the assessment. Her chief complaint was fear of dying for the past six months. She was in her usual state of mental health until she started to have sudden, unexpected attacks of fear that were associated with palpitations, shortness of breath, cold extremities, nausea, abdominal pain, lightheadedness, and fear of dying. It was also associated with a sense that the world is “odd and unreal.” These symptoms last for 15-20 minutes. She had a total of three panic attacks and experienced anticipatory anxiety between each attack. Her last panic attack was four months ago.

Before the first panic attack, the patient was complaining of persistent abdominal pain, primarily localized to the epigastric area. It was associated with bloating but no nausea, vomiting, or any other gastrointestinal symptoms. The pain was exacerbated by stress and consumption of food. However, she could not specify any dietary triggers. She underwent labs and examinations in a private clinic, and she was found to have a positive test for *H. pylori *infection. She was successfully treated with a course of two antibiotics, clarithromycin and metronidazole, and a proton pump inhibitor, omeprazole, for two weeks. She was also scheduled for an endoscopy, for which she did not show up. Three weeks later, she repeated the stool test for *H. pylori*, and the stool report was negative for *H. pylori* antigen.

Upon screening for anxiety disorders, she gave a history suggestive of illness anxiety disorder. The patient reported a preoccupation with having or acquiring a serious illness. She gave examples by saying that if she has a headache, she gets worried that it might be due to a serious disease in her brain. Medical records also show that the patient presented to the family medicine clinic with a fear of having a heart disease because she had mild chest pain. She underwent a 12-lead electrocardiogram (ECG). When the family physician reassured her that her ECG was normal, she cried because she believed that her heart disease had not been detected yet and the results could be wrong. Similarly, in another visit to a family physician, the patient reported a concern of having colon cancer. Upon inquiry, she mentioned that this concern was due to watching multiple videos about colon cancer. She did not have any family history of colon cancer, but she did mention the death of her 30-year-old cousin three years ago due to cancer. Moreover, she reported the death of multiple family members such as her grandmother and uncle as a possible trigger for her worry about death. Overall, the patient reported the need for repeated medical checkups, even after reassurance that she was fine. It is important to note that all the patient’s anxiety symptoms and panic attacks appeared after the symptoms and detection of *H. pylori* infection.

The patient reported sudden bouts of crying and previous death wishes, but she denied any suicidal or homicidal ideation, or any other depressive symptoms, such as low mood, anhedonia, sleep disturbances, decreased concentration, or psychomotor agitation. She also denied mania symptoms, visual or auditory hallucinations, delusions, and substance abuse. She did not have a family history of psychiatric disorders. Moreover, she was never on any psychiatric medications. She is single and lives with her family.

The mental status examination revealed a young female who looks her stated age. She was wearing an abaya and looked well-groomed. She was cooperative, calm, and made appropriate eye contact. There was no obvious psychomotor abnormality. Her speech was coherent and relevant with average amount, rate, and tone. She described her mood as “good” and her affect was congruent with her stated mood. Her thought process was normal and linear. There was no suicidal or homicidal ideation. No delusions, obsessions, or hallucinations were reported. She was alert, and oriented to time, place, people, and events. Her judgment and insight were fair. She refused to start medications, but she was willing to follow-up with a psychotherapist. Her lab investigations are shown in Table 1.

**Table 1:** Lab investigations of the patient. TSH: thyroid-stimulating hormone; HbA1c: glycated hemoglobin; HDL: high-density lipoprotein; LDL: low-density lipoprotein; MCV: mean corpuscular volume; RCDW: red cell distribution width

Variables	Results	Normal range
TSH	1.47 mIU/L	0.6-5.8 mIU/L
HbA1c	4.4%	3.9-6.1%
Cholesterol	4.96 mmol/L	5.18 mmol/L
HDL	1.64 mmol/L	1.55 mmol/L
LDL	2.93 mmol/L	<2.59 mmol/L
Triglyceride	0.86 mmol/L	<1.70 mmol/L
Fasting glucose	4.8 mmol/L	3.9-5.8 mmol/L
Sodium	140 mmol/L	135-144 mmol/L
Potassium	4.1 mmol/L	3.5-4.9 mmol/L
Chloride	106 mmol/L	101-111 mmol/L
Calcium	2.18 mmol/L	2.10-2.55 mmol/L
Phosphorus	1.26 mmol/L	0.8-1.44 mmol/L
Blood urea antigen	3.8 mmol/L	1.9-5.7 mmol/L
Creatinine	57 µmol/L	50-74 µmol/L
Hemoglobin	13.8 g/dL	11.5-16.5 g/dL
Hematocrit	42.7%	40-54%
MCV	89.3 fL	76-96 fL
RCDW	14.6%	11.5-14.5%
B12	337 pmol/L	129-577 pmol/L

The patient’s lab investigations were all within normal range. A diagnosis of illness anxiety disorder was made. Generalized anxiety disorder (GAD) and panic disorder were ruled out as the patient’s anxiety symptoms and panic attacks are better explained by IAD. A session with a psychotherapist was booked. Furthermore, a reschedule for endoscopy was made. The diagnosis and plan were explained to the patient, and her concerns were addressed. During the last follow-up six months later, the patient mentioned that she is still preoccupied with thoughts of having a serious illness, mainly cancer. However, these thoughts are decreasing in frequency and intensity with time. She was not experiencing abdominal pain anymore. Medical records showed that she did not show up for her psychotherapy or endoscopy appointments. Upon inquiry, she reported not showing up or canceling multiple endoscopy appointments because her friend told her that endoscopy causes sore throat, and she was worried that she would not be able to breathe. She refused to receive any education about endoscopy and mentioned that she is afraid of discovering a serious illness after endoscopy.

## Discussion

Illness anxiety disorder (IAD), previously known as hypochondriasis, is defined as the preoccupation with having or acquiring a serious illness, in the absence of somatic symptoms (or, if present, symptoms that are only mild in severity) for at least six months. It is also important to confirm that the illness is not better explained by other mental disorders [14]. According to the Diagnostic and Statistical Manual of Mental Disorders, Fifth Edition (DSM-V), patients with IAD are easily alarmed by their health status, and they experience persistent, high-level anxiety or fear of having or acquiring a serious illness, adversely affecting their daily lives. They also remain unsatisfied with their physician’s reassurances. Thus, they engage in excessive health-related behaviors (e.g., repeated checks on the body for signs of illness) or exhibit maladaptive avoidance (e.g., avoiding doctor appointments and hospitals) [14].

There are two types of IAD. The first one is the care-seeking type, and the second is the care-avoidant type [14]. In the case of our patient, it is not clear which type she is, as she frequently visited different physicians and underwent multiple tests, but at the same time, she avoided her endoscopy appointment three times due to fear of getting diagnosed with a life-threatening illness. It is also possible that the patient does not believe that the test will detect her illness, which she expressed previously after her ECG due to chest pain.

In most cases, the cause of IAD remains unclear. However, several risk factors have been documented in the literature. These include an underlying anxiety disorder, a history of previous serious illness, illness of the patient’s caregivers or family members, excess research about health-related materials, or discussions that involve the pathologization of normal body sensations [1,14]. In the present case, the patient’s history reveals many of these risk factors. Experiencing a positive test for *H. pylori *antigen and undergoing eradication therapy is believed to be the biggest risk factor in this case, as all her symptoms began after the detection of *H. pylori *infection. Another risk factor is spending an excessive amount of time reviewing serious illnesses, such as when the patient suspected she had colon cancer after watching multiple videos about it. Lastly, death and serious illness of family members were also reported in the present case. Evidence regarding the development of IAD after death of family members is scarce in the literature. However, a study showed that 15.8% of people with a vicarious experience of cancer reported clinically significant health anxiety [15].

The focus of treatment in IDA is helping the patient manage the anxiety related to their illness. Recognizing the patient's anxiety and terror is important. However, avoid superfluous referrals, unnecessary laboratory tests, and imaging studies once major medical problems have been ruled out and IAD has been diagnosed. In order to reduce the number of emergency room visits, patients should be scheduled for routine follow-up appointments with both their primary care physician and their psychiatrist [16].

The cornerstone of care for individuals with IAD is cognitive behavioral therapy (CBT). To our knowledge, no CBT subtype has yet demonstrated superiority. Still, numerous studies support its efficacy in the management of IAD [17]. Medications can also supplement IAD treatment. Randomized controlled trials support efficacy of selective serotonin reuptake inhibitors (SSRIs) in treating IAD, though they are less effective than psychotherapy [18]. Case reports suggest that TCAs, such as clomipramine, may also be helpful [19]. Healthcare providers should be aware that excessive focus on medications to treat IAD may actually worsen anxiety. For instance, patients might misread prescribing medications as an attempt to dismiss their unexplained symptoms, as well as the side effects of these medications. Patients should be reassured about using medications and given very detailed information about the management plan during their scheduled visits [20].

In the present case, the patient has not attended her psychotherapy appointment. She also has not agreed to take any psychiatric medications. However, she reported a reduction in her anxiety symptoms and panic attacks once the eradication of *H. pylori* was successful. We believe that CBT would have helped the patient more and provided her with the tools to control and cope with her anxiety symptoms. Finally, we acknowledge the limitations of our study, such as the single-case nature of the study, and the confounding factors of the development of IAD.

## Conclusions

This study is about a 21-year-old female patient with a new-onset illness anxiety disorder (IAD) after *H. pylori* infection. In this study, we covered the clinical features of IAD and discussed its diagnosis, epidemiology, and psychopathology. Furthermore, we highlighted the possible triggering factors for our patient. Finally, we discussed the evidence-based psychological and pharmacological treatments that can significantly alleviate symptoms. We suggest that family medicine physicians be aware of IAD and other mental disorders when treating patients with *H. pylori* infection as it is associated with mental health issues. Good history-taking and communication between the patient and the mental health practitioner are essential for overall disease treatment outcomes.
